# Kin discrimination drives territorial exclusion during *Bacillus subtilis* swarming and restrains exploitation of surfactin

**DOI:** 10.1038/s41396-021-01124-4

**Published:** 2021-10-14

**Authors:** Barbara Kraigher, Monika Butolen, Polonca Stefanic, Ines Mandic Mulec

**Affiliations:** 1 Chair of Microbiology, Department of Food Science and Technology, Biotechnical Faculty, University of Ljubljana, 1000 Ljubljana, Slovenia; 2 Chair of Micro Process Engineering and Technology COMPETE, University of Ljubljana, 1000 Ljubljana, Slovenia

**Keywords:** Microbial ecology, Biofilms

## Abstract

Swarming is the collective movement of bacteria across a surface. It requires the production of surfactants (public goods) to overcome surface tension and provides an excellent model to investigate bacterial cooperation. Previously, we correlated swarm interaction phenotypes with kin discrimination between *B. subtilis* soil isolates, by showing that less related strains form boundaries between swarms and highly related strains merge. However, how kin discrimination affects cooperation and territoriality in swarming bacteria remains little explored. Here we show that the pattern of surface colonization by swarming mixtures is influenced by kin types. Closely related strain mixtures colonize the surface in a mixed swarm, while mixtures of less related strains show competitive exclusion as only one strain colonizes the surface. The outcome of nonkin swarm expansion depends on the initial ratio of the competing strains, indicating positive frequency-dependent competition. We find that addition of surfactin (a public good excreted from cells) can complement the swarming defect of nonkin mutants, whereas close encounters in nonkin mixtures lead to territorial exclusion, which limits the exploitation of surfactin by nonkin nonproducers. The work suggests that kin discrimination driven competitive territorial exclusion may be an important determinant for the success of cooperative surface colonization.

## Introduction

Interactions between microbes strongly shape microbial communities and multicellular structures such as biofilms or swarms [1–5]. The nature of interactions between microbes in multicellular settings, where cells differentiate into specialized cell variants by coordinated activity and group into sedimentary biofilms or moving swarms, is usually cooperative. The cells work together as a team to build a highly adapted system protected from the environment, where they share secreted molecules (e.g. enzymes, surfactants, matrix components), which are referred to as public goods [6–8]. On the other hand, competition for different life resources between strains is also widespread, as evidenced by the fascinating variety of strategies that bacteria have developed in order to be efficient in warfare with their neighbors. Competitive strategies can either be indirect competition for resources or direct interference competition through active chemical warfare involving antimicrobial secretions or the contact-dependent killing of neighboring cells [9–12].

When one cell encounters another, which mechanisms determine the nature and outcome of their interaction? One of the possible explanations for preferential association and cooperation with some and harming and exclusion of others can be explained by kin discrimination, which is broadly defined as differential treatment of conspecifics according to their relatedness [13–15]. Kin discrimination is a widespread behavior detected among animals and plants [16, 17]. However, evidence exists that microbes have also evolved kin discrimination, which has been studied in natural populations of different microbial species [9, 18–23].

We have recently shown that 39 environmental isolates of the gram positive *Bacillus subtilis* from soil microscale (1 cm^3^) could be classified into 12 kin groups, which were designated according to the boundary phenotype between two swarms encountering each other on the semisolid agar plate [22]. This sympatric strain collection was previously subjected to analyses that demarcated strains into three ecotypes, which are ecologically distinct phylogenetic groups [24] and three pherotypes, which are groups of strains with distinct quorum sensing specificity, able to exchange signals and induce quorum sensing response within but not between groups [25]. An extensive pairwise analyses of swarm interaction phenotypes between *B. subtilis* isolates revealed that highly related (kin) isolates (>99.5% identity at the level of housekeeping genes) more frequently merged their swarms in contrast to less related (nonkin) isolates (<99.5% identity between housekeeping genes), which formed a dramatic boundary at the encounter of their swarms on soft agar [22]. Recent work shows that kin discrimination between *B. subtilis* isolates involves the combined action of different genetic loci that either encode enzymes involved in cell wall synthesis and polysaccharide matrix, transcriptional regulators of stress response and the attack and defense molecules [26, 27], but the mechanisms responsible for the antagonistic interactions between *B. subtilis* isolates are not well understood. When kin isolates were inoculated into liquid medium at 1:1 ratio and given an overnight opportunity to colonize the *Arabidopsis thaliana* plant roots, they formed mixed biofilms, while nonkin strains formed biofilms consisting primarily of one strain [22]. This experiment suggested an antagonistic interaction resulting in competitive exclusion among nonkin isolates when forming biofilms. However, the interactions between isolates in mixtures if given the opportunity for rapid surface migration by swarming from a common nutrient patch remained unknown.

Biofilms are surface aggregates of sessile bacteria with slow expansion driven by a self-produced extracellular polymer matrix [28], while swarming enables rapid coordinated surface translocation of actively self-propelled flagellated microbial population [29]. To perform rapid collective migration, cells first need to attain sufficient density in order to produce surfactants necessary for surface spreading. Once this criterion is met, cells form physically highly intimate but also highly dynamic associations [29–34]. Wild isolates of *B. subtilis* that are capable of surface spreading on 0.7% agar medium need to secrete surfactin [35], which is produced by enzymes encoded in the *srfA* operon [36–38]. Surfactin is a lipopetide antibiotic and surfactant that promotes surface spreading by reducing the surface tension [35, 39–41]. Spatial expansion of the population by swarming involves sharing of public goods (surfactants) [29, 42] and is therefore considered an example of bacterial cooperative behavior [43]. On the other hand, population expansion could be seen as competition for nutrients and space, where the better migration of cells, together with the inhibition of genetically different cells, is an advantage. Swarming thus represents an excellent model process to experimentally test social evolution theories on cooperation and competition [34].

The theory predicts that cooperative behaviors are preferentially directed towards kin [44] which has been extensively discussed by many scientists [8, 15, 45–49]. How cooperative bacteria limit exploitation by noncooperators, which can destabilize cooperation, is still a research topic of considerable interest. We here use the term “exploitation” to refer to any degree of absolute gain to a noncooperator from interacting with a cooperator, even if that gain does not lead to a relative fitness advantage of the noncooperator. It is not known whether kinship influences exploitation of surfactin by surfactin nonproducers (noncooperators) in mixed swarms. Various strategies have been proposed to limit the spread of noncooperators [43, 50–55]. For example, Ho et al. [55] suggested that by shaping the spatial structure of bacterial communities kin discrimination can limit the spread of noncooperators during cooperative swarming. We lack information on spatial structuring in a swarm when mixtures of kin or nonkin *B. subtilis* isolates are given the opportunity to colonize the swarming agar from a common patch, and whether the initial ratio of each genotype affects the territorial expansion outcome.

Although results on roots [22] suggested competition between nonkin, the dynamics and frequency dependence of this competition have not been addressed yet. Firstly, it is possible that the dominant strain outcompetes the other, regardless of the initial ratio between two strains because it grows faster, swarms faster or more efficiently kills the competitor. For example, Budding et al. [56] found that the dominant ‘Dienes type’ of *Proteus mirabilis* colonized the surface even when the nondominant strain in the mixture was inoculated at a frequency 100–10,000-fold higher than the dominant strain. Secondly, competitive interactions may involve frequency-dependent effects [57–60], which can be detected by changing the initial ratio between two strains. If an isolate at low initial frequency outcompetes the other, this represents a negative frequency-dependent competition, which has been shown for *E. coli* strains with specific trade-offs in growth rate and motility differences [61]. In contrast, positive frequency-dependent interference competition, with the winning strain being present at higher initial frequency, was found in *Myxococcus xanthus* [59].

We here investigated the role of kin discrimination in shaping the population spatial structure during swarming of *B. subtilis* soil isolates for which we previously identified kin type [22]. The effect of kinship and the initial genotype ratio on cell association and the territorial expansion by swarming was tested by mixing kin and nonkin strain pairs in different ratios and then following their assortment in developing swarms. Results confirm cell associations between kin and positive frequency-dependent competitive exclusion between nonkin. Furthermore, we demonstrate that nonkin exclusion limits the sharing of surfactin between nonkin strains.

## Materials and methods

### Strains and media

Strains of *B. subtilis* isolated from soil from the sandy bank of the river Sava in Slovenia [25] were used in this study. The strains were always first streaked from the frozen cultures onto LB agar (35 g Sigma LB broth with agar per L). All the strains are listed in Table S1. To visualize interactions and the spatial distribution of cells, the *B. subtilis* strains were fluorescently labeled using two fluorescent proteins, YFP and mKate, expressed from two constitutive promoters, P_hypercIo3_ and P_hyperspank_ respectively. To exclude the possible fluorescent protein marker effects on the results, experiments were performed in both combinations of two strains with swapped fluorescent markers. The strains were constructed by transformation of wild-type strains with DNA derived from NCIB 3610, with the desired fluorescent markers kindly provided by R. Kolter and the *B. subtilis* strains with *hagΩTn10 (Sp)* and *hag::kan’ (Kn)* constructs kindly provided by D. Kearns and A. Kovacs respectively. Appropriate antibiotics were used for strain constructions and experiments at the following concentrations: 5 μg/ml of chloramphenicol (Cm), 100 μg/ml of spectinomycin (Sp), 5 μg/ml of kanamycin (Kan), 0.5 μg/ml of erythromycin (Ery) and 12.5 μg/ml of lincomycin (Lin) for selection for *mls* resistance. Swarming assays were performed on swarming agar (final agar concentration of 0.7%), based on B-medium as described in [22].

### Experimental setup and swarming assay

Mixtures of strains were prepared from liquid monocultures and their swarming pattern after competition on the swarming agar plate was analyzed. Two self, two kin and three nonkin strain combinations with the focal PS-216 strain were tested. Their phylogenetic relatedness based on four concatenated housekeeping genes [22], ecotype and pherotype strain affiliations [24] are listed in Table 1. Additionally, we assessed the influence of surface colonization by swarming on the size of population on the agar surface by cell counts. We also tested the possibility to restore swarming of the swarming deficient mutants (*srfA* mutants) by mixing them with surfactin-producing cells or just by conditioned medium prepared from surfactin-producing cells. The ability of different *B. subtilis* isolates to colonize the surface in monocultures and mixed cultures was tested on freshly prepared 7-cm swarming agar plates containing B-medium with 0.7% agar. Strains were inoculated from overnight LB plates into 3 ml of LB-medium and shaken overnight at 37 °C. Overnight cultures were then diluted 100 times in fresh LB-medium and grown for 2.5 h to reach the early exponential phase. This procedure was repeated twice and then OD600 (optical density at 600 nm) was measured by spectrophotometer (Photometer MA9510, Iskra, Slovenia). When the OD600 reached ~0.2–0.4, a total of 2 μl of exponential phase cells (app. 10^4^–10^5^ cells) were spotted in the center of the agar surface, either as monocultures or as a mixture of two strains with different fluorescent labels at different ratios (4:1, 1:1, and 1:4). The plates were incubated for 18–20 h at 37 °C (at a humidity level of 70–80%) and then photographed. Each swarming (competition) experiment was repeated at least three times with three replicates for each experiment.

**Table 1:** Summary of tested strain combinations and their phylogenetic distance.

Focal strain	Mix partner	Ecotype identity	Pherotype identity	Nomen-clature	Phylogenetic distance
PS-216	PS-216	PE10	168	Self	0
	PS-18	PE10	168	Kin	0.001
	PS-68	PE10	168	Kin	0.001
	PS-209	PE22	RO-H-1/RO-B-2	Nonkin	0.005
	PS-196	PE22	RS-D-2/NAF4	Nonkin	0.010
	PS-218	PE32	RS-D-2/NAF4	Nonkin	0.008

Data retrieved from refs. [22, 24].

### Visualization of the fluorescent strains

The swarms of fluorescent strains were visualized at 8-times magnification using a fluorescent stereomicroscope (CH9435, type DFC425 C, Leica Microsystems, Wetzlar, Germany) equipped with filter sets ET mCherry MZ10 with excitation filter ET560/40 nm and emission filter ET630/75 nm and ET YFP MZ10 with excitation filter ET500/20 nm and emission filter ET535/30 nm for mKate and YFP fluorescent proteins, respectively. Composite images were created by the ImageJ program (National Institute of Health, Bethesda, MD, USA).

### Quantitative analyses of cell numbers in a colony or swarm

*B. subtilis* strains in the exponential phase (monocultures or mixtures) were inoculated (2 μl) on B medium with 0.7% or 1.5% agar plates at 37 °C (at a humidity level of 70–80%) for 18–20 h, as described above. All cells in the swarms or colonies were removed from the agar plate using a sterile surgical blade and then suspended in 10 ml of physiological saline solution by vigorous vortexing. Then, the sonication method to disperse cell aggregates was performed as described in Špacapan et al. and Dogsa et al. [62, 63]. The suspensions were kept on ice and 1 ml was sonicated with an amplitude of 15 μm three times for 5 s each time using an MSE 150 Watt MK2 ultrasonic disintegrator at 20 kHz (MSE Scientific Instruments, Sussex, England) in order to disintegrate cell clusters observed in the suspension and under the microscope. Cells in the sonicated suspension were serially diluted and plated on LB agar plates with appropriate antibiotics. Visible colony counts were conducted after 16 h of incubation at 37 °C. The effect of sonication on cell counts was tested by counting and comparing the cell numbers of two monocultures (wild-type and *srfA* mutant) that were either exposed or not exposed to sonication (Fig. S1).

### Statistical analyses

All experiments were repeated at least three times with three replicates in each experiment. For swarming competition outcome and pattern visualization single images of representative agar plates of kin and nonkin strain combinations are shown. For the quantitative analysis of cells in swarms or colonies the average number of cells and the standard deviations of the three replicate plates were calculated for each of the three biological replications. Two-tailed unpaired *t*-tests were performed to test for differences in cell numbers.

## Results

### Exclusion of nonkin restricts association to kin

**Fig. 1:**
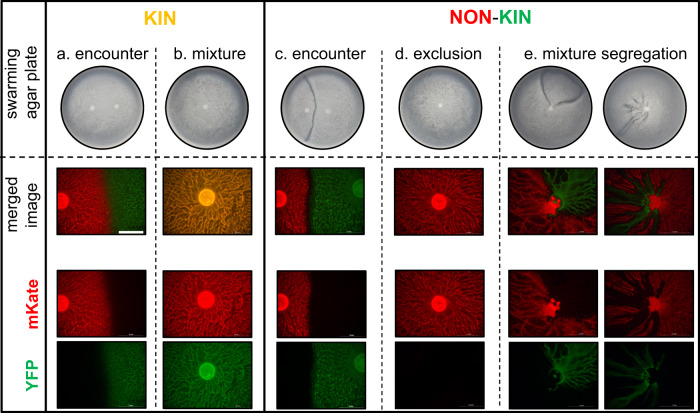
Swarm patterns of separately spotted and mixed kin and nonkin strains. In encounter experiments (**a**, **c**), two kin or nonkin strains in the exponential growth phase were spotted on the swarming agar at a distance of 3–4 cm. In the mixture experiments (**b**, **d**, **e**) strains were mixed in liquid cultures (ratio 1:1) before spotting in the center of the swarming agar. The kin combination presented here is PS-218 mKate and PS-218 YFP (**a**, **b**), and the nonkin combination is PS-218 mKate and PS-216 YFP (**c**, **d**, **e**), which are representative of all the kin and nonkin strain combinations tested. Photographs of the 7-cm agar plates are shown at the top, and at the bottom, enlarged areas of the center of each plate are shown with merged and separate YFP and mKate fluorescent image captures taken by fluorescent stereomicroscope; magnification ×8, scale bar = 5 mm.

We investigated whether the initial interactions between *B. subtilis* isolates in the inoculation drop on agar, before they engage in cooperative swarming, affect the outcome of surface colonization and the spatial distribution of cells in a swarm. To do this, we tested three nonkin, two kin and two self (differentially labeled but otherwise isogenic strains) strain combinations (Tables 1 and S2), which had been previously characterized by a swarm encounter assay [22] (Fig. 1a, c). The differentially labeled strains were mixed in equal amounts and then inoculated in the center of the swarming agar. Kin and self-mixtures colonized the surface together forming a common swarm with both fluorescent markers visible in the same dendrites, as indicated by the orange–yellow color of the swarm in the merged image (Fig. 1b). In contrast, nonkin strains, which form a prominent boundary upon swarm encounter (Fig. 1c), did not mix when inoculated together. Usually, only one strain colonized the agar (Fig. 1d), with the other strain occasionally detected only in small patches at the inoculation site. The dominant colonizer varied between experiments testing the same strain combinations, suggesting that the tested strains have similar colonizing ability and that only a slight advantage in the time-point of swarming initiation can have a profound effect on surface colonization success. In some experiments, the nonkin mixtures segregated into separate, clearly defined sectors (Fig. 1e). Although easily spotted, these lines of segregation between nonkin swarms were rare and less dramatic than in the encounter assay (Fig. 1c, e). It is important to note that we have never observed sectoring between kin swarms, which underlines the conclusion that nonkin fail to associate during cooperative movement and do not form a collective swarm.

### Relative population sizes in the swarming inoculum influence the competition outcome

**Fig. 2:**
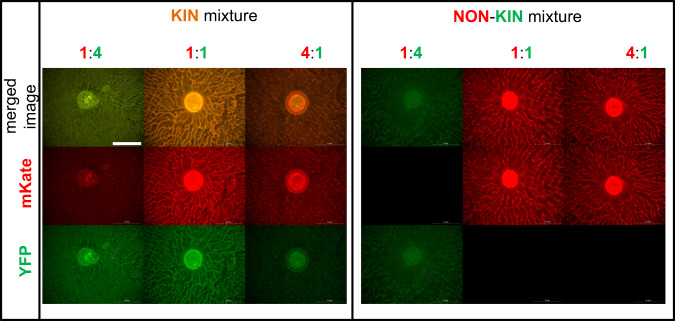
Influence of the initial ratio between strains on the colonization of swarming agar. Mixtures of kin strains (strains PS-218 mKate and PS-218 YFP are shown here) or nonkin strains (strains PS-218 mKate and PS-216 YFP are shown here) in different ratios were spotted (2 µl) in the center of the swarming agar plates and visualized by flourescent stereomicroscope. In kin mixtures of all ratios (4:1, 1:1, and 1:4) both strains swarmed together over the entire agar surface, as indicated by the yellowish color (green–yellow or orange) in the merged image. In nonkin mixtures, in most cases only the more abundant strain in the inoculum mixture colonized the surface, indicated in the merged image by either red or green. Black panels under either mKate or YFP filters mean that the specific fluorescent proteins were not detected by fluorescent stereomicroscope, indicating a significant decrease in the abundance of the corresponding strain; magnification ×8, scale bar = 5 mm.

As we have found that the dominant strain outcome in the swarming competition assays with 1:1 nonkin mixtures varied between experiments, we next tested whether the initial ratio between the two strains in the inoculum mixture affects the competition outcome. Mixtures of nonkin and kin strains were prepared in the ratios of 4:1, 1:1, and 1:4. Regardless of the initial ratio, self and kin strains (self-mixtures PS-216 with PS-216*, PS-218 with PS-218*, and kin mixtures PS-216 with PS-18* and PS-216 with PS-68*; asterisk (*) indicates a different fluorescent marker) co-colonized the agar surface (Fig. 2, Table S2). In contrast, in nonkin mixtures (PS-216 with PS-218*, PS-216 with PS-196*, and PS-216 with PS-209*), the more abundant strain in the initial inoculum excluded the other strain and colonized the complete swarm area (Fig. 2, Table S2). When the ratio between nonkin strains was 1:1, sometimes one strain dominated, and sometimes the other, suggesting that a small advantage in the initial ratio may be sufficient for one strain to gain dominance and prevent the other nonkin strain from colonizing the surface. These results further support the conclusion that kin cells associate during cooperative swarming, whereas nonkin strains exclude each other through positive frequency-dependent competition.

### Swarming deficient mutants (*srfA*) can be complemented by surfactin from kin and nonkin strains

**Fig. 3:**
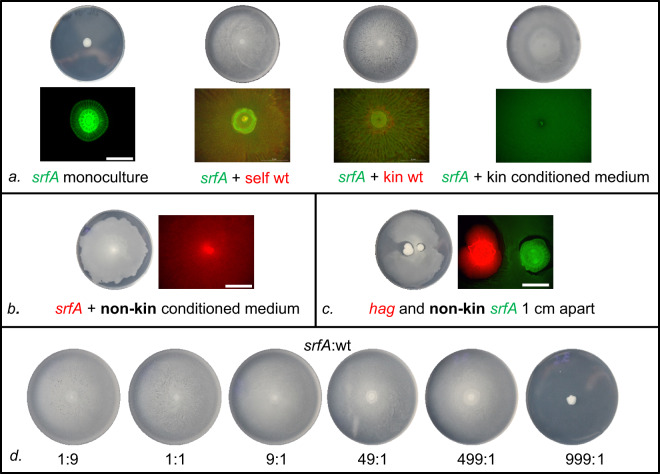
Swarming deficient mutants (*s*rfA) can be complemented by surfactin from kin and nonkin strains. **a** Surfactin from a kin strain restores swarming of the *srfA* mutant. PS-216 YFP *srfA* mutant was inoculated in the center of the swarming agar either alone or mixed with the PS-216 mKate (self-combination), with the PS-68 mKate (kin combination), or with 10 µl conditioned medium from the PS-216 mKate. When the conditioned medium was added, swarming of the mutant was restored but the pattern of swarming changed. Photographs of the 7-cm agar plates are shown at the top, and at the bottom, enlarged areas in the center of each plate are shown with merged YFP and mKate fluorescent images. Magnification ×8, scale bar = 5 mm. **b**, **c** Surfactin from the nonkin strain restores swarming of the *srfA* mutant. **b** The addition of 10 µl of conditioned medium from the nonkin wild-type strain (PS-218 YFP) promoted swarming of the PS-216 mKate *srfA* mutant. **c** Surfactin diffusing from the nonkin PS-218 mKate *hag* mutant was used by the PS-216 YFP *srfA* mutant spotted at 1-cm distance. **d** Swarming of the *srfA* mutant can be restored by a low proportion of wild-type kin cells. Strains PS-216 *srfA* and PS-216 were mixed in different proportions. Wild type restored swarming with only 0.2% in the initial mix.

Surfactin is considered a public good, which is freely shared during swarming. It has been shown previously that the isogenic *srfA* mutants of *B. subtilis* that do not produce surfactin and therefore fail to swarm alone are incorporated by the wild-type parental strain into a common swarm [35]. To test this with our selected strains we first mixed the surfactin producer *B. subtilis* PS-216 and its isogenic *srfA* mutant and confirmed complementation of the mutant by the wild type. As expected, the mutant alone did not swarm, but when mixed with its kin producer PS-216 it swarmed over the entire plate (Fig. 3a). The same results were obtained for the PS-218 *srfA* mutant and its wild-type parental strain (Table S3), and when two kin (but not isogenic) strains PS-216 *srfA* mutant and the wild-type PS-68 were mixed (Fig. 3a). Moreover, conditioned medium prepared by growing the wild-type strain in LB liquid medium and spotted on the agar complemented the swarming defect of its *srfA* mutant (Fig. 3a). We also prepared conditioned medium by filtering nonkin wild-type strains grown to the stationary phase and then mixed it with the *srfA* mutant cells. The surfactin in the conditioned medium complemented the swarming of the *srfA* mutant, regardless of whether it originated from a kin or nonkin culture (Fig. 3a, b), suggesting that surfactin could be exploitable when produced by nonkin cells growing in immediate vicinity, but this is not likely due to nonkin competitive exclusion. The results also indicated that monocultures grown in the liquid medium did not extracellularly accumulate any compounds that would act antagonistically against nonkin and suggested that kin discrimination requires direct cell-to-cell contact in order to achieve antagonistic interactions between nonkin. To test the prediction that kin discrimination depends on cell-to-cell interactions between nonkin, we first investigated the long-distance interactions between the nonkin *srfA* and the *hag* mutants, neither of which can swarm, as the former has flagella but lacks surfactin and the latter actively produces surfactin but lacks flagella [35]. The two mutants were spotted at different distances on the same swarming plate. The nonkin surfactin producer promoted swarming of the *srfA* mutant, when the two strains were spotted at a distance of 0.5–1 cm (Figs. 3c and S2), confirming that without physical cell-to-cell contact the *hag* mutant can share surfactin even with the nonkin.

Before testing the effect of close nonkin interactions on public good sharing we determined the minimum proportion of wild-type cells required to restore swarming of the *srfA* mutant. We mixed different proportions of the wild type and the mutant. Merely 0.2% of wild-type cells in the inoculum mixture was sufficient to support swarming of the mutant (although this proportion was higher when the fluorescent labels were switched; Figs. 3d and S3), suggesting that only a minor portion of surfactant producing cells in a population is sufficient for cooperative swarming. Moreover, we tested whether the *srfA* mutant could gain a relative fitness advantage by exploiting the goods produced by the surfactin-producing parental strain in the swarming mixture. The results indicated an increase in relative abundance of the *srfA* mutant as compared to its wild-type parent in 1:1 inoculation mixes that could not be explained by fluorescent marker effects (Fig. S4), which suggests that the mutant could potentially pose a threat to cooperation.

### Exclusion of nonkin affects the sharing of surfactin during swarming

**Fig. 4:**
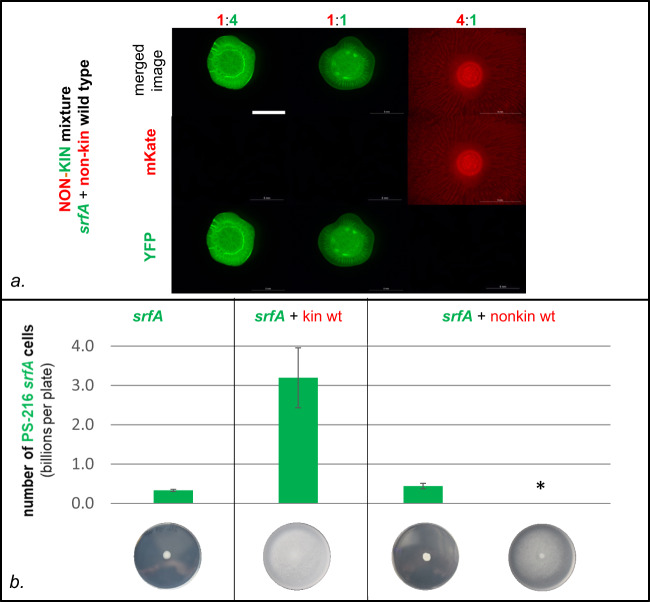
Sharing of surfactin is affected by frequency-dependent competitive exclusion of nonkin. **a** When the mutant PS-218 YFP *srfA* was mixed with the nonkin wild-type PS-216 mKate in different ratios (4:1, 1:1, and 1:4), the winning strain was the more abundant one. Swarming of the mutant was not rescued by the wild type at any ratio tested. Enlarged areas of the center of the plates are shown with merged and separated YFP and mKate fluorescent images; magnification ×8, scale bar = 5 mm. The experiment was repeated in three biological replicates (each with three technical replicates). Representative results of the experiments are shown. **b** Population size of the *srfA* mutant strain increases when it cooperates with the kin wild type, but not when mixed with the nonkin wild type. The PS-216 *srfA* YFP cells (2 µl) were mixed with 2 µl of sterile B medium or 2 μl of kin (PS-216 mKate) or nonkin strains (PS-218 mKate) and spotted in the center of the agar. After overnight incubation, the number of PS-216 *srfA* YFP cells in each colony was determined. The average number of three experiments (each represented by three replicate plates) with standard deviations are shown. A significant increase (*P* < 0.01) in the number of PS-216 YFP *srfA* cells was only detected when the mutant was mixed with kin wild-type cells. When PS-216 YFP *srfA* was mixed with the nonkin wild-type cells, one of two scenarios occurred: either the mutant conquered the plate and did not swarm, or the nonkin wild type conquered the plate and the mutant cell counts were less than 10^5^ (*). Photographed 7-cm agar plates are shown at the bottom. * when the competing nonkin strain PS-218 mKate colonized the swarming plate, the number of PS-216 *srfA* YFP cells was lower than 10^5^, which is less than 0,01% of the total population.

Next, we investigated the sharing of surfactin during swarming in genetically mixed cultures. Considering the surface exclusion observed between nonkin strains, we predicted that the sharing of surfactin would be absent or highly restricted in nonkin mixtures. The results of testing nonkin wild-type and *srfA* mutant mixtures confirmed our prediction as the swarming was not restored when the *srfA* mutant excluded the wild-type strain (Fig. 4a). This experiment was performed with the strains PS-216 and PS-218 in all possible combinations (with one or the other strain carrying *srfA* mutation and with switched fluorescent markers). In a total of 75 independent experiments we never observed complementation of the mutant, regardless of its initial frequency (Table S3). If the mutant was inoculated with a higher initial ratio of 4:1 (*srfA*: wt), complementation also did not occur (Fig. 4a), because the mutant excluded the nonkin surfactin producer at the inoculation site. This suggested that the competitive success of the strain in mixed cultures was not dependent on the production of surfactin and its ability to swarm, but was rather governed by the initial ratio between the competing nonkin cells in the inoculum. We can conclude that the mutant was not able to exploit the nonkin surfactin producer because of the frequency-dependent territorial exclusion.

Additionally, we counted the number of *srfA* mutant cells per swarming agar plate when the mutant was in monoculture or mixed with the kin or nonkin wild-type strain. When the swarming of the *srfA* mutant was supported by surfactin produced by the kin strain, the expansion increased the number of *srfA* mutant cells per plate by 10-fold as compared to the number of cells in the non-swarming colony (Fig. 4b). On the contrary, when the *srfA* mutant was mixed with the nonkin wild-type strain, the cell counts of the *srfA* mutant were either very similar as in the non-swarming monoculture *srfA* mutant or, in the case when nonkin wild-type strain dominated on the plate, the *srfA* mutants were not detected (they represented less than 0.01% of the total population, Fig. 4). Similar increase in cell counts were obtained for the monocultures of the swarming PS-216 wild type when compared to the *srfA* mutant or to the wild type grown on a higher agar percentage, which prevented swarming (Fig. S5).

**Fig. 5:**
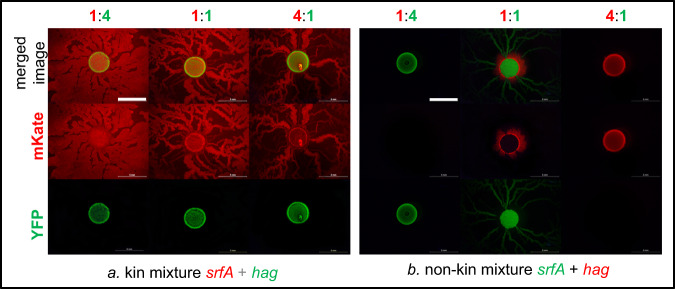
Flagella defective mutant *hag* can help *srfA* mutant to swarm. **a** *hag* mutant can restore swarming of the kin *srfA* mutant. When two kin non-swarming mutants, PS-216 YFP *hag* and PS-216 mKate *srfA*, were mixed, the *srfA* mutant could swarm in all the ratios tested. **b** Swarming is restricted in nonkin *hag* and *srfA* mixtures. When nonkin mutants (PS-218 YFP *srfA* and PS-216 mKate *hag*) were mixed in different ratios, weak swarming occurred only if both strains survived (at a strain ratio of 1:1). Magnification ×8, scale bar = 5 mm.

According to our experiments, territorial exclusion prevented cooperation between nonkin, which was confirmed by the cell counts of the *srfA* mutant which were not increased when mixed with the nonkin wild type. We further tested this by mixing the *srfA* and *hag* mutants in the swarming competition assay. In this setting, the *hag* mutant served as a potential altruistic surfactin provider to the *srfA* mutant, which could not colonize the surface alone. As expected from earlier work on *B. subtilis* NCIB 3610 by Kearns and Losick [35], in self-combination the PS-216 *srfA* mutant successfully colonized the agar surface with the help of the PS-216 *hag* mutant, which remained trapped at the inoculum spot (Fig. 5a, Table S4). Surprisingly, in this setting the PS-218 *srfA* mutant sometimes managed to escape from the nonkin PS-216 *hag* mutant by swarming on the waves of the mutant’s surfactin, although the colonization efficiency by the “escaper” was visibly reduced as compared to the kin mixtures (Fig. 5b). This happened in ~40% of the experiments where nonkin mutants were inoculated at a ratio of 1:1 and in ~8% of the experiments at ratios 1:4 and 4:1 (Table S4). Consistent with previous results, when the ratio between the strains was 4:1, in most cases the dominant strain in the initial inoculum inhibited growth of the other strain, thus preventing complementation of the nonkin *srfA* mutant by the surfactin producer (Fig. 5b, Table S4). This suggests that the outcome of competition between mutants was governed by the positive frequency-dependent competitive exclusion. On the contrary, cooperative behavior between kin was confirmed in kin mixtures, as swarming of the PS-216 *srfA* mutant was always complemented by the PS-216 *hag* mutant in all three ratios tested (Fig. 5a and Table S4).

## Discussion

We show here that kin discrimination limits the cooperative movement of *B. subtilis* strains by excluding nonkin cells from a common swarm before swarming is initiated. Although surfactin produced by the nonkin strain could restore swarming of the *srfA* mutant, the territorial exclusion between nonkin prevents the effective exploitation of this public good. Furthermore, the competition outcome indicates the positive frequency-dependent relationship, as the strain that is initially more abundant succeeds to colonize the entire agar surface. Nonkin combinations of *B. subtilis* strains showed a comparable chance to win when mixed in equal amounts, which is in contrast with the results obtained by Budding et al. [56] and Gude et al. [61] who showed that in mixed bacterial populations the strain at lower initial frequency could outcompete the other. However, consistent with our results, positive frequency-dependent interference competition has also been indicated for nonkin *M. xanthus* isolates during fruiting body formation, and diversity in patchily structured communities has been predicted to be promoted by positively frequency-dependent selection [59].

Cooperation selectively directed towards relatives could be achieved through mechanisms of kin discrimination [44]. In the previous kin discrimination swarming assay [22], strains were first allowed to spread over the territory as monocultures, and interactions between the two were limited to the narrow contact zone, where further expansion of both strains was prevented by spatial constraints and killing between nonkin strains as indicated by live/dead cells staining and by scanning electron microscopy micrographs of the lysed cells at the boundary [27, 64]. In this work, we applied a swarming competition assay, in which two exponentially growing strains were first mixed in a liquid culture and then inoculated onto the swarming agar, thus being exposed to different ecological conditions and selective pressures than in the encounter assay. In the inoculation drop cells have nutrients available to grow and competition between nonkin strains could be performed by chemical weapons. One of the possible mechanisms of killing the close nonkin could be mediated by WapAI toxin delivery system, which was found to be one of potential determinants of combinatorial kin discrimination system in *B. subtilis* [27], but further studies are needed to corroborate importance of this toxin in co-swarming assays. Alternatively, the cells could escape the close nonkin interactions by colonizing a new territory first if they start swarming faster. As we have already observed in the previous study [22], some degree of territoriality on the semisolid medium is maintained simply by the “first come, first served” rule—meaning that the already occupied territory could not be reoccupied even by the kin cells when they came to the territory later. Therefore, quicker feeding or more rapid growth and expansion by one strain could be a mechanism of competitive exclusion that we have observed. However, we demonstrate that a slight change in the initial strain frequency results in switched winning strain, suggesting that the two competing strains have very similar growth rates and expansion abilities. Furthermore, expansion by swarming is only possible if the cells cooperate and form cell groups, so-called rafts that can together migrate outwards, away from antagonistic interactions with the competing nonkin strains. This gives the cells a double advantage: they avoid fighting with the nonkin strains and by expansion they gain new nutrients and space for growth. However, to be capable of swarming, they must first grow to the population density required to produce sufficient surfactin to overcome the physical constraints posed by surface tension [41]. Results based on the assay applied in this work show that in most cases only one strain succeeds to spread over the entire territory, while the other remains trapped at the inoculation point. These, along with earlier results [27, 64], strongly support the existence of antagonistic behavior between nonkin strains. However, it is also possible that the strain at higher initial frequency limits the spreading of nonkin strain because it colonizes the surrounding territory first, hence the killing mechanism may not be always driving the competition outcome between nonkin. Moreover, the conclusions of this study are based on three nonkin, two kin and two self-strain combinations. To examine the generality of our findings more broadly, further studies with more combinations of strains would provide additional support for our conclusions.

The most extensively tested strain combination of nonkin strains, PS-216 and PS-218, belong to different pherotypes [25], which means that they do not exchange the pheromones needed to activate the production of surfactin. Signaling between nonkin strains is therefore not synchronized, which is necessary for the cooperative swarming process to occur. Our results support the conclusion that a very small difference in the initial cell density between the competing strains could have a profound effect on the outcome of the competition during a swarming assay. It is possible that a strain which first attains the required quorum density and then transforms its physiological state to active swarming would have a temporary advantage, although this still needs to be tested directly. Specialized swarmer cells form in thin films at the extreme edge of the swarm and then cells rapidly spread across the surface [32, 65, 66], which suggests that only a short time of temporary advantage is sufficient to occupy the nearby territory around the inoculation point. This prevents the other strain from colonizing the surface, as there is no longer an unoccupied surface around the inoculation drop. The swarming segregation events observed when two nonkin strains were mixed at an initial ratio of 1:1 could be explained by the effect of the local strain frequency on the outcome of the competition for the surface. Influence of local frequencies on the competition outcome between *M. xanthus* strains was demonstrated by an experiment, where each subpopulation patch was initiated with one competitor in a 99:1 majority at spatially separated inoculation spots and at each patch the more abundant strain was the winner [59]. Rendueles et al. [59] conclude that positively frequency-dependent selection maintains diversity when genotype frequencies vary patchily in structured populations.

Surfactin is a public good required for swarming in *B. subtilis*, and by using the *hag* and *srfA* mutants, neither of which can migrate alone, we show that surfactin is sharable even between nonkin strains. Surfactin produced by nonkin supports the swarming of the *srfA* mutant if the mutant is not in physical contact with the producer, suggesting that the social molecule surfactin is functionally conserved across divergent conspecifics. This extends the results of a somewhat different comet assay used by Lyons and Kolter [26], who showed that the exploitability of surfactin is possible between closely related strains that belong to different kin types. Moreover, we find here that a very low percentage of wild-type PS-216 cells (0.2%) mixed with the *srfA* mutant is sufficient to support swarming. This is consistent with the finding of Lyons and Kolter [50], who showed that PS-216 produces far more surfactin than is required for swarming.

In conclusion, we show that territorial exclusion occurs between nonkin conspecifics but not between kin. Moreover, results support the hypothesis that competitive exclusion prevents the sharing of public goods among the less related. The work uncovers that kin discrimination contributes to the maintenance of cooperative behavior and could shape the diversity of microbial communities through frequency-dependent competition for surfaces.

## Supplementary information

Supplementary Information
