# Supplementary material for: Kin discrimination drives territorial exclusion during Bacillus subtilis swarming and restrains exploitation of surfactin
Source: ISME J. 2021 Oct 14;16(3):833–41. doi: 10.1038/s41396-021-01124-4 (PMC8857193; doi:10.1038/s41396-021-01124-4)
Supplement: Supplementary file 1 — Supplementary Information [file 41396_2021_1124_MOESM1_ESM.pdf]

ISME J

Supplementary Information

Kin discrimination drives territorial exclusion during *Bacillus subtilis* swarming and restrains exploitation of surfactin

Barbara Kraigher, Monika Butolen, Polonca Stefanic and Ines Mandic Mulec

Biotechnical Faculty, University of Ljubljana, 1000 Ljubljana, Slovenia

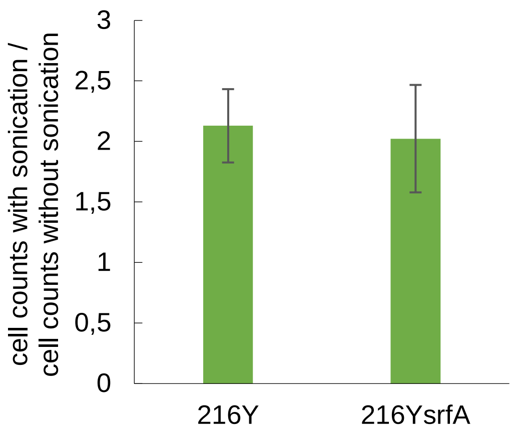

Fig. S1. Influence of sonication treatment on the cell counts. Monocultures of PS-216 YFP (216Y) and PS-216 YFP *srfA* mutant cells (216Y*srfA*) were spotted (2 µl of cells in the exponential phase) on the 1.5% agar and after overnight incubation cell counts were performed without (before) and with (after) the sonication treatment, which was applied to disperse the aggregated cells observed in the suspension and under the microscope. An increase in the cell counts was detected after the sonication treatment as compared to no treatment (indicating dispersion of cells by sonication). No significant differences between the two tested strains were detected in the ratio between cell counts with and without the sonication. Average ratios between the cell counts with and without the sonication from three replicate plates for each strain are shown. Error bars represent standard deviations.

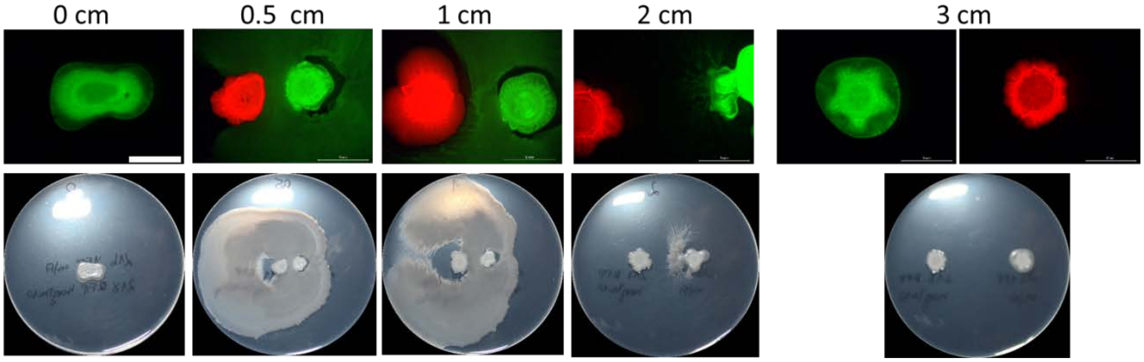

Fig. S2. Swarming of the *srfA* mutant can be promoted by the nonkin producer of surfactin lacking flagella when spotted 0.5 - 1 cm away from the *srfA* mutant. The nonkin fluorescent mutant strains PS-218 mKate *hag* and PS-216 YFP *srfA* were inoculated at different distances from each other, as indicated. Photos of 7-cm agar plates (bottom) and enlarged merged fluorescent images of both strains (top) are shown; magnification ×8, scale bar = 5 mm.

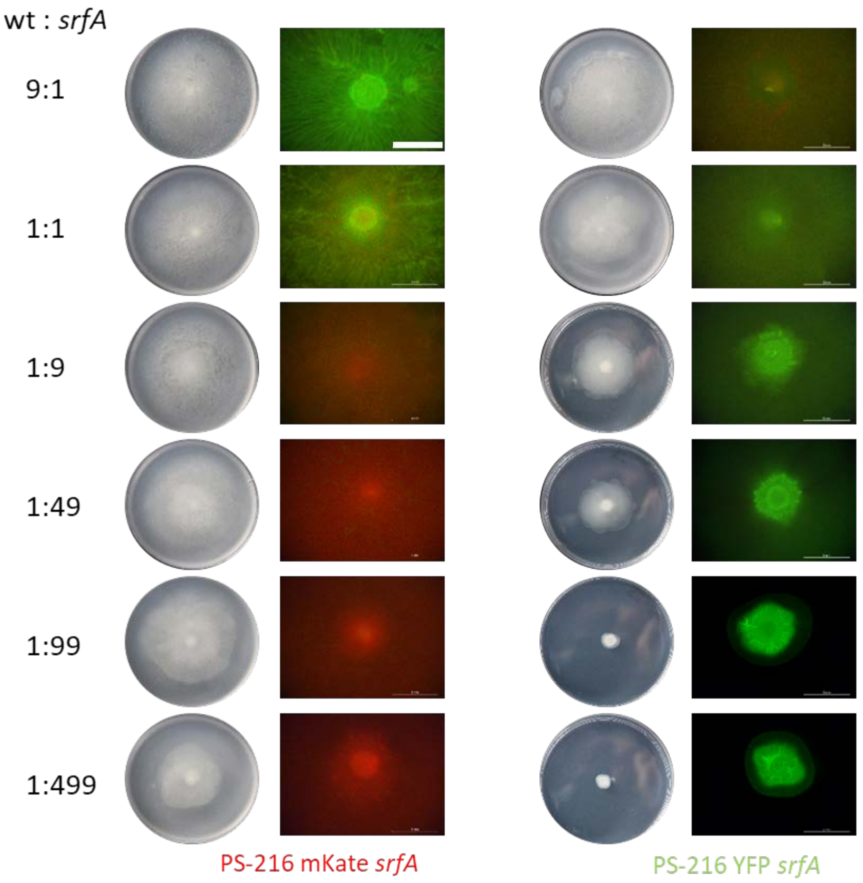

Fig. S3. Swarming of the *srfA* mutant can be restored by a small proportion of wild type kin cells. Florescent strains (PS-216 mKate *srfA* with PS-216 YFP and PS-216 YFP *srfA* with PS-216 mKate) were mixed in different proportions, swarming was still restored with only 0.2% - to 2% of the fluorescent wild type strain in the initial mix. Photos of 7-cm agar plates (left) and enlarged merged fluorescent images (right) are shown; magnification ×8, scale bar = 5 mm.

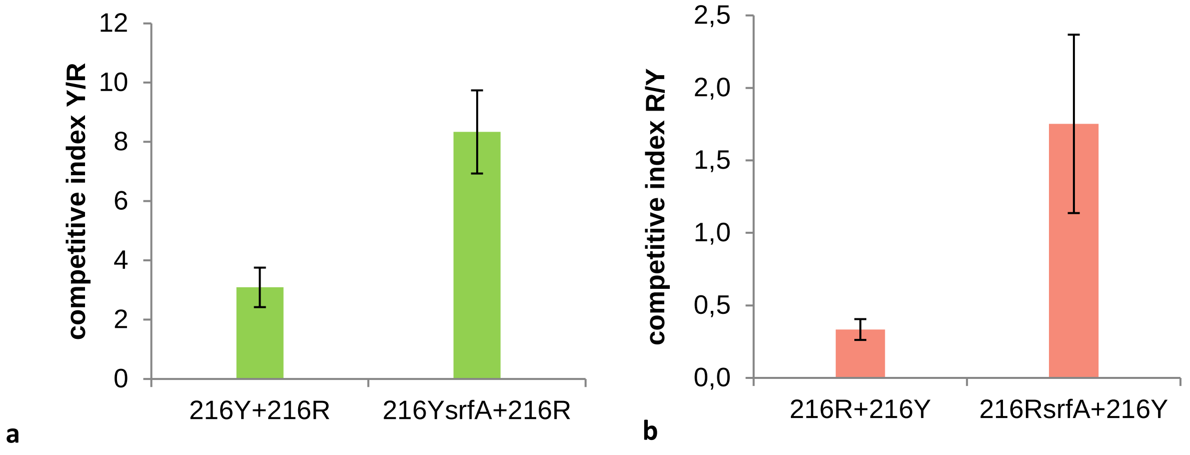

Fig. S4. *SrfA* mutant can use surfactin from its parent wild type strain to efficiently colonize the surface and increase its relative frequency in the swarming mixture. The wild type PS-216 mKate cells (216R) were mixed with the wild type PS-216 YFP (216Y) or with the PS-216 YFP *srfA* mutant cells (216Y*srfA*) in approximately 1:1 ratio and spotted in the center of the 0.7 % agar. After overnight incubation, the numbers of the two differentially labeled cells in each swarm were determined by cell counts and the ratio between the numbers of the two strains was divided by initial ratio in the mixture. A significant increase (P<0.01) in competitive index (CI, defined as the output ratio of the two strains in a mixture divided by their input ratio) between PS-216 YFP *srfA* and PS-216 mKate cells as compared to the CI between two wild type cells (PS-216 YFP and PS-216 mKate) was detected (a). Although a 3-fold increase in the ratio between two wild type strains was consistently detected (CI for Y/R was approximately 3), which was attributed to slight differences between the two fluorescent markers, the CI of the mutant and the wild type was significantly higher (a; P=0.004). Comparable advantage of the mutant was observed also with swapped fluorescent markers (b; P=0.023). The average CI values for the two mixed strains in the swarms from 2-3 independent experiments with 3 replicate swarming plates for each strain combination are shown. Error bars represent standard deviations.

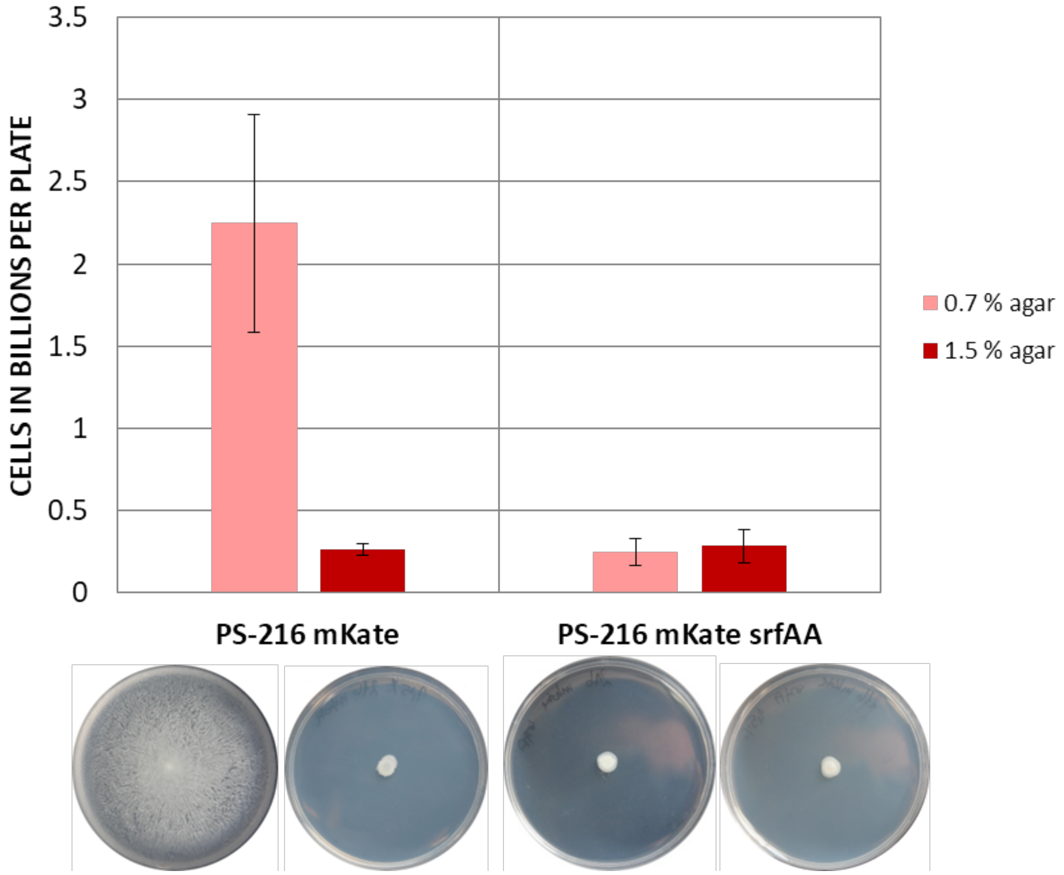

Fig. S5. Surface colonization by swarming increases population size of wild type *B. subtilis*. The wild type PS-216 mKate (wt) and the PS-216 *srfA* mKate mutant, which does not produce surfactin, were inoculated in the center of 0.7 or 1.5 % agar and allowed to swarm overnight. Cells were then removed from the surface using a sterile surgical scalpel blade and counted by CFU after sonication. Preventing cells from swarming by a high agar % (1.5%) or by *srfA* mutation reduced the number of cells grown on the plate ($P < 0.002$). Experiments were performed in three biological replicates with three technical replicates. Average values with standard deviations are shown.

Table S1. Strains used in this study

Strain code	*B. subtilis* strain name	Genetic background	Reference /source
CY49		*NCIB 3610 amyE*::P*hyperspank*-*mKate2* (Cm)	(1)
PB133		NCIB 3610 *amyE*::Phyperclo3-*yfp* (spec)	(2)
DS1143		NCIB 3160 *hag::Tn10* (spec)	(3)
PB5250		*NCIB 3610 trpC2 hagΔ717* (kan)	(4)
BM1044	PS-216 srfA	*PS-216 srfA::Tn917 (mls)*	(5)
BM1090	PS-216 YFP	PS-216 *amyE*::Phyperclo3-*yfp* (Sp)	this work
BM1097	PS-216 mKate	PS-216 *amyE*::Phyperspank-*mKate2* (Cm)	(6)
BM1125	PS-218 YFP	PS-218 *amyE*::Phyperclo3-*yfp* (Sp)	this work
BM1098	PS-218 mKate	PS-218 *amyE*::Phyperspank-*mKate2* (Cm)	(6)
BM1516	PS-18 YFP	PS-18 *amyE*::Phyperclo3-*yfp* (Sp)	this work
BM1449	PS-18 mKate	PS-18 *amyE*::Phyperspank-*mKate2* (Cm)	this work
BM1518	PS-68 YFP	PS-68 *amyE*::Phyperclo3-*yfp* (Sp)	this work
BM1451	PS-68 mKate	PS-68 *amyE*::Phyperspank-*mKate2* (Cm)	this work
BM1313	PS-196 YFP	PS-196 *amyE*::Phyperclo3-*yfp* (Sp)	this work
BM1094	PS-196 mKate	PS-96 *amyE*::Phyperspank-*mKate2* (Cm)	this work
BM131	PS-209 YFP	PS-209 *amyE*:: Phyperclo3-*yfp* (Sp)	this work
BM1095	PS-209 mKate	PS-209 *amyE*::Phyperspank-*mKate2* (Cm)	this work
BM1536	PS-216 YFP srfA	PS-216 *amyE::Phyperclo3-yfp (spec) srfA::Tn917 (mls)*	this work
BM1535	PS-216 mKate srfA	PS-216 *amyE::Phyperspank-mKate2 (Cm) srfA::Tn917 (mls)*	this work
BM1547	PS-218 YFP srfA	PS-218 *amyE::Phyperclo3-yfp (spec) srfA::Tn917 (mls)*	this work
BM1548	PS-218 mKate srfA	PS-218 *amyE::Phyperspank-mKate2 (Cm) srfA::Tn917 (mls)*	this work
BM1602	PS-216 YFP hag::kan	PS-216 *amyE::Phyperclo3-yfp (spec) hagΔ717 (kan)*	this work
BM1603	PS-216 mKate hag::kan	PS-216 *amyE::Phyperspank-mKate2 (Cm) hagΔ717 (kan)*	this work
BM1549	PS-216 mKate hagΩTn10	PS-216 *amyE::Phyperspank-mKate2 (Cm) hagΩTn10 (Sp)*	this work
BM1551	PS-218 mKate hagΩTn10	PS-218 *amyE::Phyperspank-mKate2 (Cm) hagΩTn10 (Sp)*	this work

Table S2. Strain pairs tested in swarming competition experiments. Two kin, two self- and three nonkin strain combinations were tested. Kin combinations colonized the swarming area together – indicated by the orange color, while in nonkin combinations the more abundant strain in the initial mixture colonized the plate. At the ratio 1:1, the winning strain was variable or, sometimes, sectors of both strains occurred on the plate, indicated as segregation.

	strain pair	colonization outcome		
		initial ratio between the strains		
		4:1	1:1	1:4
kin (self)	PS-216 YFP + PS-216 mKate			
	PS-218 YFP + PS-218 mKate			
	PS-216 YFP + PS-18 mKate			
	PS-216 YFP + PS-68 mKate			
non-kin	PS-216 YFP + PS-218 mKate		or<br>or	
	PS-216 YFP + PS-196 mKate			
	PS-216 YFP + PS-209 mKate			
	PS-218 YFP + PS-216 mKate			
	PS-196 YFP + PS-216 mKate			
	PS-209 YFP + PS-216 mKate			

Legend:

both labeled strains (YFP and mKate) swarmed over the plate
strain labeled with YFP swarmed over the plate
strain labeled with mKate swarmed over the plate
segregation: both labeled strains swarmed in separate sectors

Table S3. Sharing of surfactin between wild type and *srfA* mutant strains in kin-self and nonkin strain combinations. Wild type and *srfA* mutant strains (marked with different fluorescent labels) were mixed in different ratios and spotted on the swarming agar. Complementation of the *srfA* mutant by surfactin sharing was detected by the ability of the *srfA* mutant to colonize the plate, which always happened when kin strains were mixed, while in the nonkin strain pairs the more abundant strain in the inoculum outcompeted the other strain, thus preventing the complementation of swarming.

	strain pair<br>wild type with *srfA* mutant	colonization outcome<br>initial ratio between the strains			number of all experiments	% of *srfA* swarming events
		4:1	1:1	1:4		
kin-self	PS-216 YFP + PS-216 mKate *srfA*	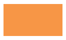	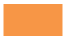	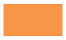	114	100
	PS-216 mKate + PS-216 YFP *srfA*					
	PS-218 YFP + PS-218 mKate *srfA*					
	PS-218 mKate + PS-218 YFP *srfA*					
non-kin	PS-216 YFP + PS-218 mKate *srfA*	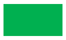	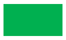 or 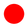	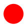	75	0
	PS-218 YFP + PS-216 mKate *srfA*					
	PS-218 mKate + PS-216 YFP *srfA*	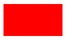	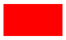 or 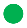	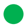		
	PS-216 mKate + PS-218 YFP *srfA*					

Legend:

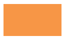 both labeled strains (YFP and mKate) swarmed over the plate

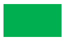 strain labeled with YFP swarmed over the plate

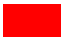 strain labeled with mKate swarmed over the plate

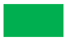 or 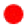 either strain labeled with YFP swarmed over the plate or strain labeled with mKate grew but did not swarm

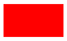 or 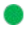 either strain labeled with mKate swarmed over the plate or strain labeled with YFP grew but did not swarm

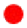 strain labeled with mKate grew but did not swarm

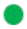 strain labeled with YFP grew but did not swarm

Table S4. Sharing of surfactin between *hag* and *srfA* mutant strains in kin and nonkin strain combinations. *Hag* and *srfA* mutant strains (marked with different fluorescent labels) were mixed in different ratios and inoculated on the swarming agar. Complementation of the *srfA* mutant by surfactin sharing was detected by the ability of the *srfA* mutant to colonize the plate, which always happened when kin-self strains were mixed, while in the nonkin strain pairs the more abundant strain in the inoculum outcompeted the other strain. Complementation of swarming was observed when both nonkin mutant strains survived on the plate, which mostly occurred when the two strains were mixed at a 1:1 ratio.

	strain pair<br>*hag* mutant with *srfA* mutant	colonization outcome<br>initial ratio between the strains		
		4:1	1:1	1:4
kin-self	PS-216 YFP *hag::kan* + PS-216 mKate *srfA*	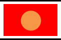	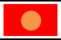	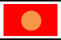
	PS-216 mKate *hag::kan* + PS-216 YFP *srfA*<br>PS-216 mKate *hagΩTn10* + PS-216 YFP *srfA*<br>PS-218 mKate *hagΩTn10* + PS-218 YFP *srfA*	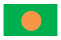	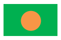	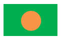
	number of all experiments	29	42	30
	% of *srfA* swarming events	100	100	100
non-kin	PS-216 YFP *hag::kan* + PS-218 mKate *srfA*	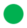	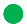 or 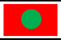 or 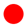	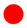
	PS-216 mKate *hag::kan* + PS-218 YFP srfA<br>PS-216 mKate hagΩTn10 + PS-218 mKate *srfA*<br>PS-218 mKate hagΩTn10 + PS-216 mKate *srfA*	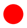	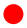 or 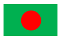 or 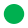	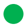
	number of all experiments	36	48	36
	% of *srfA* swarming events	8.3	39.3	8.3

Legend:

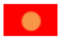 both labeled strains (YFP and mKate) grew but only mKate swarmed over the plate

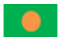 both labeled strains (YFP and mKate) grew but only YFP swarmed over the plate

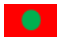 strain labeled with mKate swarmed over the plate, while YFP grew only at the inoculum spot

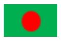 strain labeled with YFP swarmed over the plate, while mKate grew only at the inoculum spot

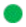 strain labeled with YFP grew but did not swarm

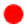 strain labeled with mKate grew but did not swarm

Supplemental references

1. Chen Y, Cao S, Chai Y, Clardy J, Kolter R, Guo J-h, *et al*. A *Bacillus subtilis* sensor kinase involved in triggering biofilm formation on the roots of tomato plants. Mol Microbiol. 2012;85(3):418-30.

2. Lyons NA, Kraigher B, Stefanic P, Mandic-Mulec I, Kolter R. A combinatorial kin discrimination system in *Bacillus subtilis*. Curr Biol. 2016;26(6):733-42.

3. Kearns DB, Chu F, Branda SS, Kolter R, Losick R. A master regulator for biofilm formation by *Bacillus subtilis*. Mol Microbiol. 2005;55(3):739-49.

4. Senesi S, Ghelardi E, Celandroni F, Salvetti S, Parisio E, Galizzi A. Surface-associated flagellum formation and swarming differentiation in *Bacillus subtilis* are controlled by the *ifm* locus. J Bacteriol. 2004;186(4):1158-64.

5. Oslizlo A, Stefanic P, Vatovec S, Beigot Glaser S, Rupnik M, Mandic-Mulec I. Exploring ComQXPA quorum-sensing diversity and biocontrol potential of *Bacillus* spp. isolates from tomato rhizoplane. Microb Biotechnol. 2015;8(3):527-40.

6. Stefanic P, Kraigher B, Lyons NA, Kolter R, Mandic-Mulec I. Kin discrimination between sympatric *Bacillus subtilis* isolates. Proc Natl Acad Sci U S A. 2015;112(45):14042-7.
